# Epigenetic silencing of microRNA-137 enhances ASCT2 expression and tumor glutamine metabolism

**DOI:** 10.1038/oncsis.2017.59

**Published:** 2017-07-10

**Authors:** J Dong, D Xiao, Z Zhao, P Ren, C Li, Y Hu, J Shi, H Su, L Wang, H Liu, B Li, P Gao, G Qing

**Affiliations:** 1 Department of Pharmacology, School of Basic Medicine, Tongji Medical College, Huazhong University of Science and Technology, Wuhan, China; 2 Department of Cancer Biology, Medical Research Institute, Wuhan University, Wuhan, China; 3 Clinical Laboratory, The Second Hospital of Dalian Medical University, Dalian, China; 4 School of Pharmacy, Hubei University of Science and Technology, Xianning, China; 5 Department of Biochemistry and Molecular Biology, Zhongshan School of Medicine, Sun Yat-Sen University, Guangzhou, China; 6 Union Hospital, Tongji Medical College, Huazhong University of Sicence and Technology, Wuhan, China; 7 Affiliated Dalian Sixth People’s Hospital, Dalian Medical University, Dalian, China; 8 Department of Biotechnology, Dalian Institute of Chemical Physics, Dalian, China

## Abstract

Tumor cells must activate specific transporters to meet their increased glutamine metabolic demands. Relative to other glutamine transporters, the ASC family transporter 2 (ASCT2, also called SLC1A5) is profoundly elevated in a wide spectrum of human cancers to coordinate metabolic reprogramming and malignant transformation. Understanding the molecular mechanisms whereby tumor cells frequently upregulate this transporter is therefore vital to develop potential strategies for transporter-targeted therapies. Combining *in-silico* algorithms with systemic experimental screening, we herein identify the tumor suppressor microRNA, miR-137, as an essential regulator that targets ASCT2 and cancer cell glutamine metabolism. Metabolic analysis shows that miR-137 derepression, similar to ASCT2 inactivation, significantly inhibits glutamine consumption and TCA cycle anaplerosis. Mechanistically, methyl-CpG-binding protein 2 (MeCP2) and DNA methyltransferases (DNMTs) cooperate to promote active methylation of the miR-137 promoter and inhibit its transcription, conversely reactivating ASCT2 expression and glutamine metabolism. Moreover, expression between miR-137 and ASCT2 is inversely correlated in tumor specimens from multiple cancer types, and ectopic ASCT2 expression markedly rescued miR-137 suppression of tumorigenesis. These findings thus elucidate a previously unreported mechanism responsible for ASCT2 deregulation in human cancers and identify ASCT2 as a critical downstream effector of miR-137, revealing a molecular link between DNA methylation, microRNA and tumor metabolism.

## Introduction

Elevated glutamine metabolism is an essential feature of malignant transformation. The importance of glutamine as a global, critical nutrient in fueling proliferation and survival has become better understood and appreciated in recent years.^1, 2, 3, 4^ Glutamine provides metabolic intermediates to replenish the tricarboxylic acid (TCA) cycle and maintain the mitochondrial integrity and nicotinamide adenine dinucleotide phosphate (NADPH) levels.^5, 6, 7, 8, 9, 10, 11, 12, 13, 14, 15, 16^ In addition, its carbon skeleton can be incorporated into glucose and fatty acids while the nitrogen part is used in the biosynthesis of purines and pyrimidines.^5, 8, 9, 10, 16, 17^ As such, glutamine metabolism not only provides cancer cells building blocks into an array of growth-promoting pathways, but also alleviates them from wicked cellular microenvironment by maintaining a proper redox homeostasis.

Glutamine is hydrophilic and water soluble, hence, extracellular glutamine cannot simply diffuse into cells across the plasma membrane. Instead, intracellular glutamine levels are tightly controlled by and strictly dependent on the membrane-anchored transporter systems that differ in structure, mechanism and regulation.^18, 19^ Indeed, a significant correlation has been identified between glutamine transport and glutaminolysis.^20^ Glutamine influx is mediated by three major families of transporter systems, system A, ASC and N.^18, 19^ Among which, the ASC family member ASCT2, a high-affinity glutamine transporter, is receiving increasing attention for its critical roles in mediation of glutamine-dependent tumor cell growth, mammalian target of rapamycin (mTOR) activation and drug resistance.^21, 22, 23, 24^ Genetic depletion or pharmacological inhibition of ASCT2 results in growth repression and apoptosis in multiple human cancer types.^18, 23, 24^ ASCT2 functions as a Na^+^-dependent transporter for glutamine as well as alanine, serine and cysteine.^23, 24^ The transport process is electroneutral, involving the Na^+^-coupled influx of glutamine to the Na^+^-coupled efflux of another substrate such as alanine, serine or cysteine, thus contributing to the homeostasis of amino acid metabolism within tumor cells.

MicroRNAs (miRNAs) are a family of small, non-coding RNAs that regulate gene expression by blocking translation or promoting degradation of target mRNAs.^25^ Deregulated miRNAs can rewire multiple cellular and biological processes, contributing to initiation and progression of human cancers. MiRNAs have been implicated in regulation of multiple aspects of tumor cell metabolism including glucose metabolism, lipid homeostasis and amino-acid biogenesis.^7, 26, 27^ However, the direct links between miRNA deregulation and glutamine transport, the corresponding regulatory mechanisms as well as the clinical relevance of such mechanisms in human cancers remain unknown.

Given the essential roles of ASCT2 in glutamine-dependent metabolic reprogramming and the relevance of perturbed miRNA signaling in malignant transformation, we herein set out to screen miRNAs involved in ASCT2 regulation. We identify that miR-137, a tumor suppressor microRNA silenced in a wide spectrum of human cancers,^28, 29, 30, 31, 32^ selectively targets ASCT2 and tumor glutamine metabolism. Mechanistic studies show that MeCP2 and DNA methyltransferases cooperate to promote active methylation of the miR-137 promoter and its decreased transcription, leading to enhanced ASCT2 expression and glutamine metabolism. Analysis of sequencing data from Cancer Genome Atlas confirms that the expression levels between miR-137 and *ASCT2* are inversely correlated in multiple human cancer types. These findings thus elucidate a novel, global mechanism accounting for ASCT2 deregulation in human cancers, revealing a molecular link between miR-137, ASCT2 and tumor metabolism.

## Results

### Identification of miR-137 and miR-122 as potential regulators of ASCT2

**Figure 1:**
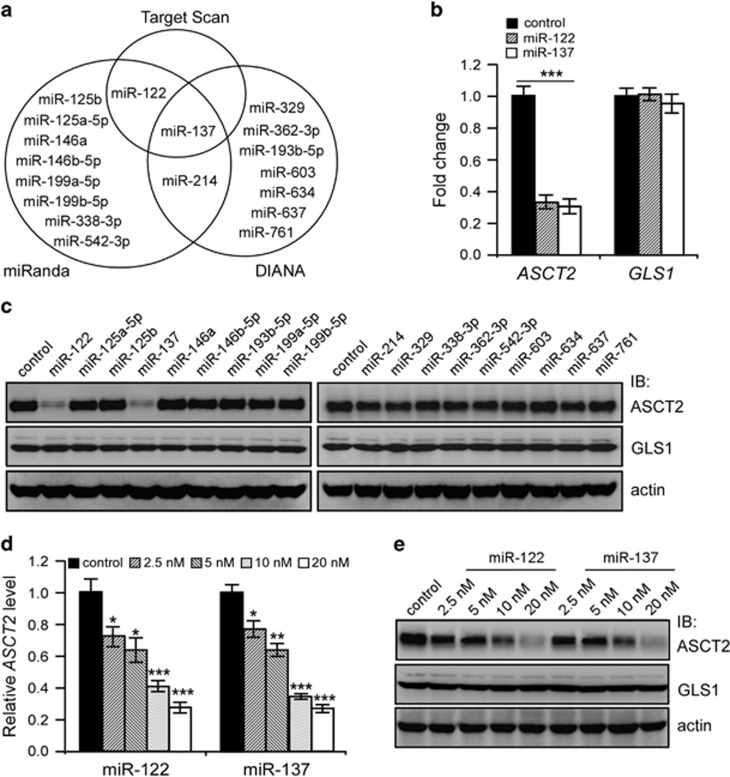
Systemic identification of miRNAs targeting ASCT2. (**a**) MiRNAs predicted to target ASCT2 by three algorithms. (**b**) Real-time qPCR quantification of *ASCT2* and *GLS1* mRNA levels in 293T cells 24 h post transfection of control, miR-137 or miR-122 mimics. Mean±s.d. (*n*=3), *t*-test; ***P<0.001. (**c**) Immunoblot analysis of ASCT2 and GLS1 protein levels in 293T cells upon transfection of indicated miRNA mimics. Actin was used as a loading control. (**d**, **e**) Ectopic expression of miR-137 or miR-122 dose-dependently reduced *ASCT2* mRNA (**d**) and protein (**e**) levels in 293T cells. Actin was used as a loading control. Mean±s.d. (*n*=3), *t*-test; *P<0.05; **P<0.01; ***P<0.001.

To investigate miRNAs potentially implicated in ASCT2 regulation, we performed *in-silico* analysis using the TargetScan (www.targetscan.org), miRanada (www.microrna.org/microrna/home.do) and DIANA (http://diana.cslab.ece.ntua.gr/microT) algorithms, and identified a total of 18 miRNAs as predicted to target the 3′-untranslated region (UTR) of *ASCT2* mRNA (Figure 1a). Notably, miR-137 was the only one identified by these three algorithms.

To identify positive miRNA hits, we respectively transfected the mimics of these miRNAs into 293T cells and analyzed ASCT2 expression 24 h post transfection. Transient transfection of either miR-137 or miR-122 mimics markedly inhibited endogenous ASCT2 expression in 293T cells, whereas the remaining mimics exhibited undetectable effects (Figure 1b and c and Supplementary Figure S1). The inhibitory effects of miR-137 or miR-122 mimics on ASCT2 appeared quite specific, as the expression of glutaminase 1 (GLS1, encoded by *GLS1*), a critical enzyme catalyzing the conversion of glutamine to glutamate, was barely affected (Figure 1b and c and Supplementary Figure S1). Further analysis showed that both miR-137 and miR-122 mimics inhibited ASCT2 in a dose-dependent manner, resulting in prominent ASCT2 reduction even at a lower dose of 2.5 nM (Figure 1d and e).

Since miR-137 functions as a global tumor suppressor miRNA in a wide spectrum of human cancers whereas miR-122 is a liver-specific miRNA downregulated in hepatocellular carcinoma,^28, 29, 30, 31, 32, 33, 34^ we focused on miR-137 regulation of ASCT2 in the current study.

### MiR-137 targets ASCT2 by directly binding to its 3′-UTR

**Figure 2:**
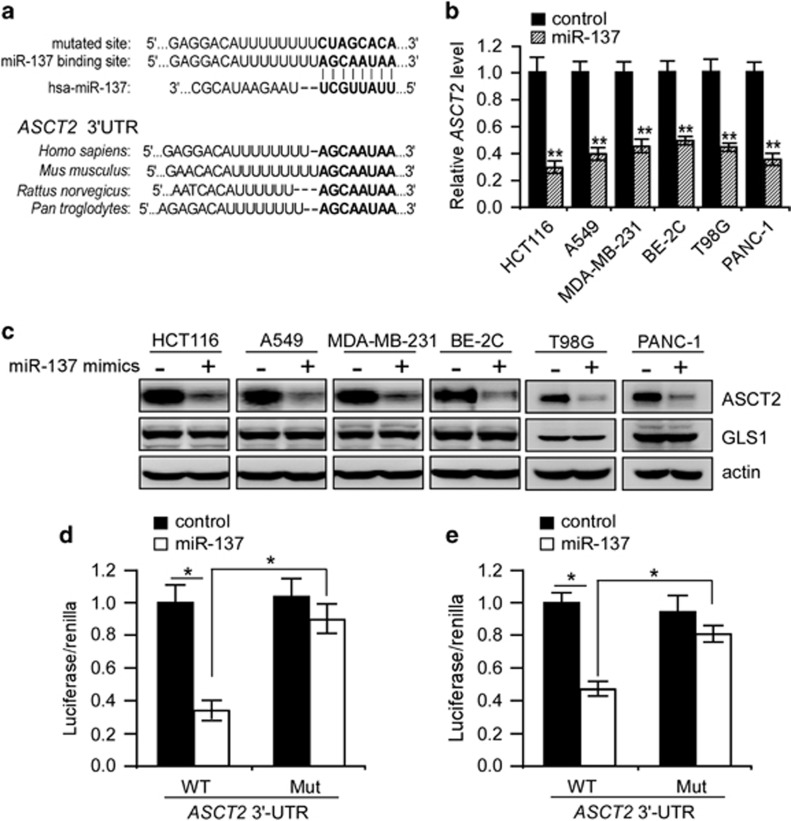
MiR-137 directly targets *ASCT2* 3′-UTR. (**a**) Base-pair binding between miR-137 and putative miRNA-binding sites within *ASCT2* 3′-untranslated regions (UTRs) across different species. (**b**, **c**) MiR-137 mimics selectively reduced *ASCT2* mRNA (**b**) and protein (**c**) levels in tumor cells from different cancer types. Actin was used as a loading control. Mean±s.d. (*n*=3), *t*-test; **P<0.01. (**d**, **e**) Luciferase assays of human *ASCT2* 3′-UTR constructs with intact and mutated seed sequences for miR-137 in 293 T (**d**) and HCT116 (**e**) cells. Mean±s.d. (*n*=3), *t*-test; *P<0.05.

The TargetScan algorithm predicts that miR-137 could selectively target the seed sequence within 3′-UTR of *ASCT2* mRNA (Figure 2a). Of note, this sequence is highly conserved across different species (Figure 2a), indicating that specific base-pairing to this site by miR-137 may have an evolutionarily conserved role in regulation of ASCT2 expression. MiR-137 is silenced in colorectal carcinoma, glioblastoma, neuroblastoma, prostate and pancreatic cancers.^28, 29, 30, 31, 32^ We therefore examined if miR-137 could decrease the ASCT2 abundance in tumor cells from these cancer types. As expected, transient transfection of miR-137 mimics consistently inhibited endogenous ASCT2 expression in all the tumor cells examined without affecting GLS1 expression (Figure 2b and c and Supplementary Figure S2a). Administration of miR-137 mimics had also undetectable effects on the expression of glycolytic genes as well as the aerobic glycolysis of HCT116 cells (Supplementary Figure S2b–d). All these data suggest that inhibition of ASCT2 expression and glutamine metabolism is important for miR-137 tumor suppressor functions.

**Figure 3:**
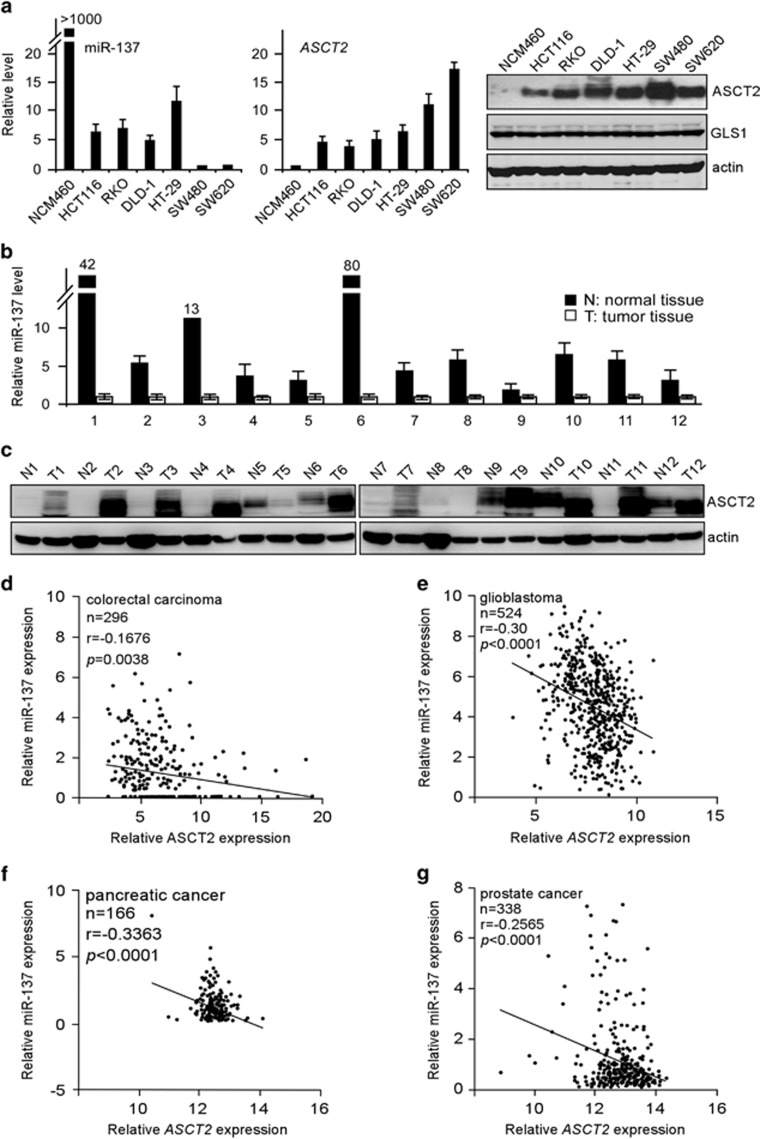
Inverse correlation between ASCT2 and miR-137 expression in clinic tumor specimens. (**a**) Relative levels of miR-137 (left panel) and ASCT2 (right panel) in normal colonic epithelial NCM460 cells and colorectal carcinoma cell lines analyzed by real-time qPCR and immunoblot assays. Actin was used as a loading control. (**b**, **c**) Relative levels of miR-137 (**b**), analyzed by real-time qPCR, and ASCT2 (**c**), analyzed by immunoblot, in twelve pairs of normal colorectal tissues and colorectal carcinoma specimens. (**d**–**g**) Correlation between miR-137 and *ASCT2* in colorectal carcinoma (**d**), glioblastoma (**e**), pancreatic (**f**) and prostate cancer (**g**), performed by Pearson’s correlation analysis.

**Figure 4:**
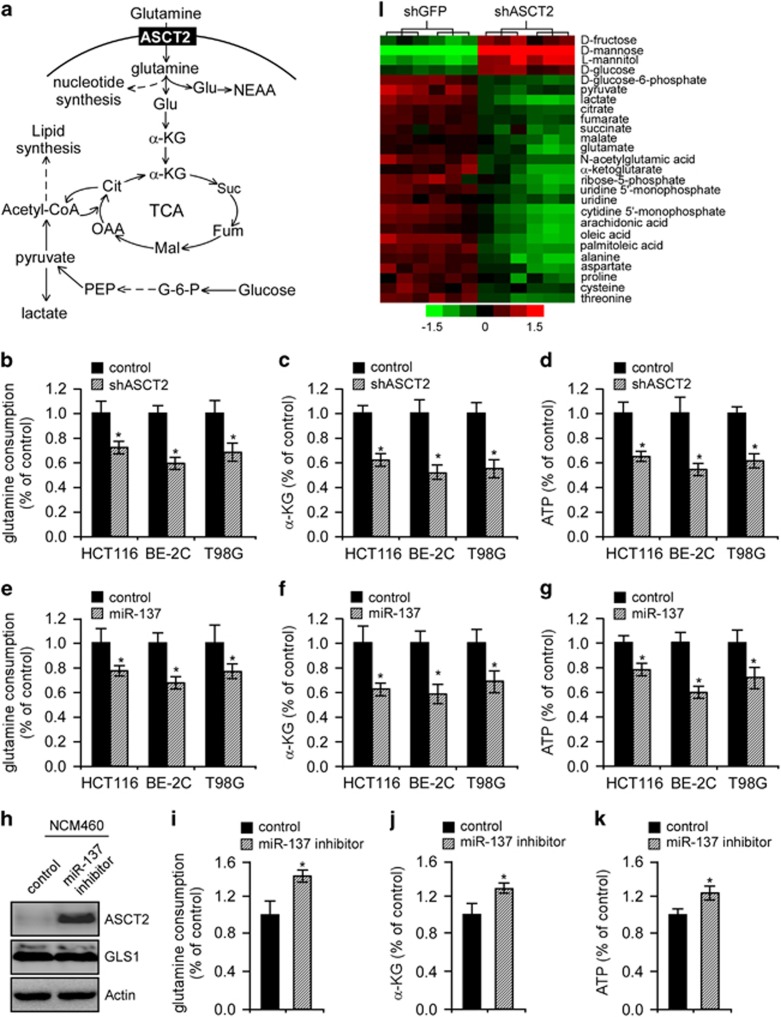
Metabolic analysis upon modulation of miR-137 or ASCT2 expression. (**a**) Schematic diagraph depicting glutamine metabolism. α-KG, α-ketoglutarate; Cit, citrate; G-6-P, glucose-6-phosphate; Fum, fumarate; Glu, glutamate; Mal, malate; NEAA, non-essential amino acids; OAA, oxaloacetate; PEP, phosphoenolpyruvate; Suc, succinate. (**b**–**d**) Relative changes in glutamine consumption (**b**), α-KG production (**c**) and intracellular ATP content (**d**) in HCT116, BE-2C and T98G cells upon ASCT2 depletion. Mean±s.d. (*n*=3), t-test; *P<0.05. (**e**–**g**) Relative changes in glutamine consumption (**e**), α-KG production (**f**) and intracellular ATP content (**g**) in HCT116, BE-2C and T98G cells upon miR-137 overexpression. Mean±s.d. (*n*=3), *t*-test; *P<0.05. (**h**–**k**) Relative changes in ASCT2 protein levels (**h**), glutamine consumption (**i**), α-KG production (**j**) and intracellular ATP content (**k**) in NCM460 cells upon depletion of endogenous miR-137. Mean±s.d. (*n*=3), *t*-test; *P<0.05. (**l**) Metabolite profiling of HCT116 cells upon ASCT2 inhibition. Relative levels of metabolites are shown in sextuplicates. The scale bar shows color-coded differential changes in metabolites. Red: metabolite abundance increased; green: metabolite abundance decreased.

We then determined whether miR-137 directly targets and inhibits the expression of ASCT2 through specific base-pair binding to its 3′-UTR. To test this, we cloned the 3′-UTR sequence of *ASCT2* including the predicted miR-137-binding site into pGL3-promoter vector. Indeed, inclusion of the *ASCT2* 3′-UTR inhibited luciferase activity when 293T or HCT116 cells were co-transfected with miR-137 mimics, whereas mutation of the predicted binding site significantly reversed inhibition of luciferase expression under similar conditions (Figure 2d and e), validating ASCT2 as a direct miR-137 target. In support of this notion, inhibition of endogenous miR-137 using an miRNA sponge significantly increased endogenous ASCT2 levels in NCM460 cells, a normal colon epithelial cell line exhibiting remarkable endogenous miR-137 expression (Figures 3a and 4h).

### ASCT2 is inversely correlated with miR-137 in multiple human cancers

To examine a potential correlation between ASCT2 and miR-137, we analyzed their expression levels in normal colonic epithelial cells (NCM460) and series of colorectal carcinoma cells (Figure 3a). Across the seven cell lines examined, we identified an inverse correlation between miR-137 expression and ASCT2 protein levels. Of note, miR-137 expression was lowest in SW480 and SW620 tumor cells, which conversely exhibited the highest ASCT2 abundance, whereas NCM460 cells with the highest miR-137 expression showed a much lower ASCT2 protein level (Figure 3a), suggesting miR-137 as an essential regulator of endogenous ASCT2 expression.

We then analyzed their relative levels in a matched collection of 12 human colorectal carcinoma specimens and non-tumor tissues. As expected, overall miR-137 expression was significantly lower in tumor samples than in adjacent normal tissues, where ASCT2 levels were conversely elevated (Figure 3b and c). These observations prompted us to investigate whether there is any inverse correlation between the expression of miR-137 and *ASCT2* in human cancers. For this, we analyzed sequencing data from Cancer Genome Atlas (http://tcga-data.nci.nih.gov/tcga/). Interestingly, miR-137 levels significantly, but conversely, correlated with *ASCT2* in multiple cancer types, including colorectal carcinoma, glioblastoma, prostate and pancreatic cancers (Figure 3d–g). We could not identify such a correlation between miR-122 and *ASCT2* in these cancer types (Supplementary Figure S3a–d). Instead, as a liver-specific tumor suppressor microRNA, miR-122 abundance was conversely correlated with *ASCT2* expression in human hepatocellular carcinomas (Supplementary Figure S3e). Altogether, these results suggest that miR-137 might play a more universal role in suppression of ASCT2 expression and glutamine metabolism.

### MiR-137 overexpression elicits similar metabolic dysfunction as ASCT2 depletion

As a critical nutrient for cancer cells, glutamine serves as a carbon and nitrogen source for biosynthesis of macromolecules and, via conversion to α-ketoglutarate (α-KG), as an ATP source through the TCA cycle and oxidative phosphorylation (Figure 4a). To assess the potential roles of ASCT2 in oxidative glutamine metabolism, we first analyzed glutamine consumption in HCT116, BE-2C and T98G cells. As expected, ASCT2 depletion significantly inhibited glutamine consumption and decreased the intracellular α-KG levels by ~40% (Figure 4b and c), suggesting that ASCT2 mediates glutamine influx to sustain TCA cycle anaplerosis in these tumor cells. Abrogation of ASCT2 expression also inhibited ATP generation (Figure 4d). Notably, ectopic expression of miR-137 similarly inhibited glutamine consumption, α-KG production and ATP generation (Figure 4e–g), whereas its depletion in NCM460 cells significantly enhanced endogenous ASCT2 abundance and glutamine consumption concomitant with a prominent increase in α-KG and ATP production (Figure 4h and k), arguing that miR-137 inhibits glutamine metabolism primarily through ASCT2 downregulation.

To more completely evaluate the ASCT2 functions, we metabolically profiled the control and ASCT2-depleted HCT116 cells by gas chromatography mass spectrometry (GC/MS; Figure 4l). Relative to the mock treatment, depletion of ASCT2 caused a marked decrease in glutamic acid, *N*-acetylglutamic acid, α-KG, L-aspartate and L-proline, all of which are downstream metabolites of glutamine metabolism. Critical TCA cycle intermediates, including citrate, fumarate, succinate and malate, were also significantly depleted. ASCT2 also regulated the concentrations of other important amino acids, such as threonine and cysteine, and essential metabolic intermediates involved in nucleotide and lipid biosynthesis, such as ribose-5-phosphate, uridine and cytidine 5′-monophosphates (for nucleotide biosynthesis) as well as arachidonic acid, oleic acid and palmitoleic acid (for lipid biosynthesis). In addition, ASCT2 depletion caused fructose and glucose accumulation concomitant with a decline in glucose-6-phosphate, pyruvate and lactate content, indicating a malfunction of aerobic glycolysis (the Warburg effect). All these metabolites are essential building blocks for tumor cell growth and proliferation.

### Ectopic ASCT2 expression partially rescued miR-137 suppression of tumorigenesis

**Figure 5:**
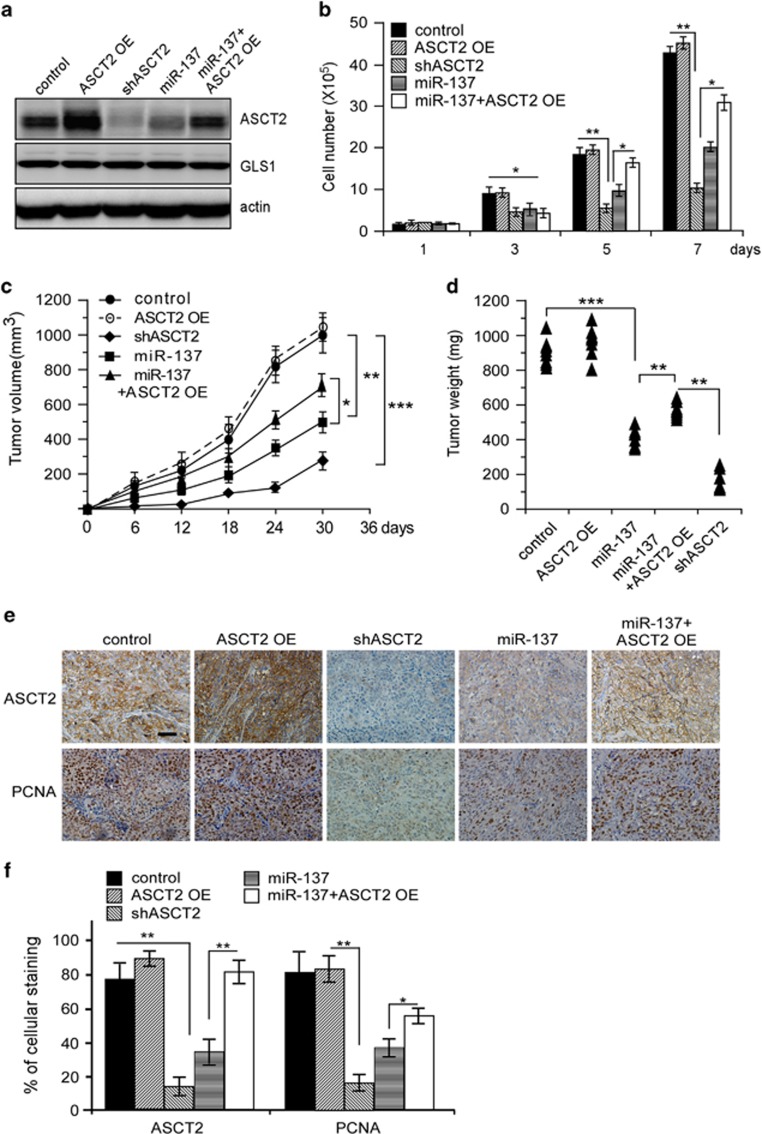
Ectopic ASCT2 expression partially reversed miR-137 suppression of tumorigenesis. (**a**) Immunoblot analysis of ASCT2 protein levels in HCT116 cells expressing the indicated plasmid constructs. Actin was used as a loading control. ASCT2 OE, ASCT2 overexpression; miR-137, miR-137 overexpression; miR-137+ASCT2 OE, both miR-137 and ASCT2 overexpression; shASCT2, ASCT2 knockdown. (**b**) *In vitro* cell proliferation of HCT116 cells expressing the indicated constructs as shown in **a**. Mean±s.d. (*n*=3), *t-*test; *P<0.05; **P<0.01. (**c**, **d**) Enforced expression of ASCT2 partially rescued HCT116 xenograft tumor growth inhibited by miR-137, as presented by tumor volume (**c**) or weight (**d**). Eight tumors were analyzed per group, *t-*test; *P<0.05; **P<0.01; ***P<0.001. (**e**, **f**) Immunostaining (**e**) and quantification (**f**) of ASCT2 and PCNA in tumor sections derived from (**d**). Eight tumors were analyzed per group, *t*-test. The scale bar represents 50 μm. *P<0.05, **P<0.01.

To examine whether inhibition of ASCT2 expression could impact the miR-137 tumor suppressor functions, we generated HCT116 cells expressing the control vector, ASCT2 [ASCT2 OE], ASCT2 shRNA [shASCT2], miR-137 or miR-137/ASCT2 combination [miR-137+ASCT2 OE] (Figure 5a). As expected, either ASCT2 shRNA knockdown or miR-137 ectopic expression significantly inhibited HCT116 proliferation, whereas ASCT2 overexpression partially, but significantly, rescued cell proliferation repressed by miR-137 (Figure 5b). Note that glutamine deprivation also significantly inhibited HCT116 cell proliferation (Supplementary Figure S4), suggesting that ASCT2’s ability to promote cell growth relies on, at least in part, increased glutamine metabolism. These cells were then, respectively, inoculated subcutaneously into nude mice, and the growth of resultant tumors was monitored. Again, mice inoculated with HCT116 cells harboring shASCT2 or miR-137 consistently developed much smaller tumors than those with the control cells, whereas restoration of ASCT2 expression significantly reversed miR-137 inhibitory effect (Figure 5c–f). Ectopic expression of ASCT2 alone had minimal effect on tumorigenesis, arguing that the high level of endogenous ASCT2 was sufficient to sustain glutamine metabolism and tumor growth. Altogether, these results support that ASCT2 downregulation contributed to miR-137 inhibition of tumorigenesis *in vivo*.

### Epigenetic silencing of miR-137 by MeCP2 and DNMTs reactivated ASCT2 expression and glutamine metabolism

**Figure 6:**
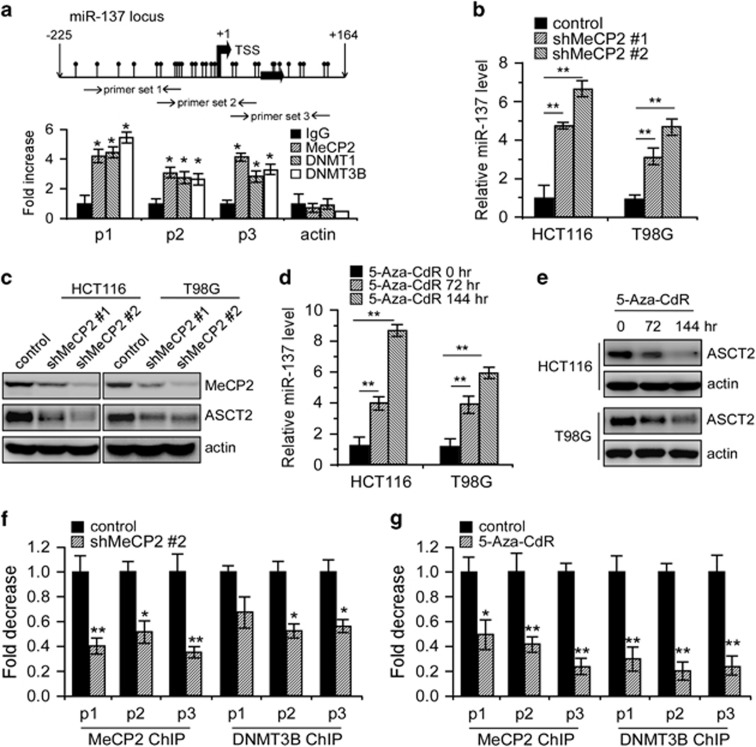
Epigenetic silencing of miR-137 by MeCP2 and DNMTs reactivated ASCT2 expression. (**a**) Binding of MeCP2 and DNMTs to the miR-137 CpG island analyzed by ChIP assay in HCT116 cells. The *ACTIN* promoter was used as a negative control. Results were presented as averages of fold changes between MeCP2, DNMT1, DNMT3B ChIP and isotype IgG controls. P1, primer set 1; P2, primer set 2; P3, primer set 3. Mean±s.d. (*n*=3), *t*-test; *P<0.05. (**b**–**e**) Effects of MeCP2 depletion or 5′-Aza-CdR treatment on miR-137 (**b**, **d**) and ASCT2 expression (**c**, **e**) in HCT116 and T98G cells. Actin was used as a loading control. Mean±s.d. (*n*=3), *t*-test; **P<0.01. (**f**, **g**) Binding of MeCP2 and DNMT3B to the miR-137 CpG island analyzed by ChIP assay in HCT116 cells upon MeCP2 depletion (**f**) or 5′-Aza-CdR treatment (**g**). Results were presented as averages of fold changes between MeCP2, DNMT3B ChIP and isotype IgG controls. Mean±s.d. (*n*=3), *t*-test; *P<0.05, **P<0.01.

**Figure 7:**
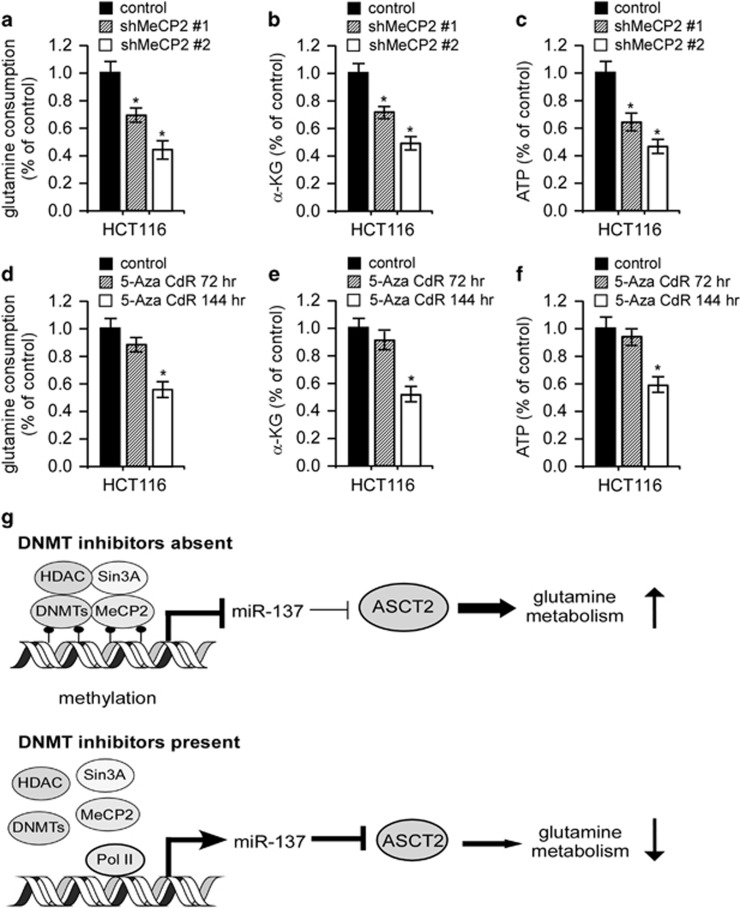
Metabolic analysis upon MeCP2 depletion or DNMTs inhibition. (**a**–**c**) Relative changes in glutamine consumption (**a**), α-KG production (**b**) and intracellular ATP content (**c**) in HCT116 cells upon MeCP2 depletion. Mean±s.d. (*n*=3), *t*-test; *P<0.05. (**d**–**f**) Relative changes in glutamine consumption (**d**), α-KG production (**e**) and intracellular ATP content (**f**) in HCT116 upon DNMTs inhibition. Mean±s.d. (*n*=3), *t*-test; *P<0.05. (**g**) A simplified model depicting ASCT2 regulation by miR-137. See text for more details.

Previous studies suggested epigenetic silencing of miR-137 involving the Polycomb Group proteins EZH2/SUZ12 and/or DNA hypermethylation.^35, 36^ Yet, the molecular mechanisms whereby miR-137 is epigenetically silenced remain to be determined. We depleted EZH2 expression in HCT116 cells, but found that its inhibition had little effect on miR-137 and ASCT2 expression (Supplementary Figure S5). MiR-137 presents a clear CpG island in its upstream and downstream chromosomal region flanking the transcriptional start site (TSS) (Figure 6a). DNA methyltransferases (DNMTs) frequently methylate the cytosine within CpG dinucleotides gathered in CpG islands. Methylated CpGs then recruit DNA-methyl CpG-binding proteins (MeCPs and MBDs).^35^ Chromatin immunoprecipitation (ChIP) assays revealed the presence of DNMT1, DNMT3B and MeCP2 at the pre-miR-137 chromatin regions harboring CpG islands, whereas no observable reactivity was detected at the *ACTIN* promoter, which was used as a negative control (Figure 6a). We next depleted MeCP2 with two specific shRNAs in HCT116 and T98G cells or treated these cells with the DNMT inhibitor 5-aza-2-deoxycytidine (5-Aza-CdR). As expected, either treatment significantly increased endogenous miR-137 expression while conversely depleted ASCT2 accumulation (Figure 6b–e). In support of this notion, either MeCP2 knockdown or 5-Aza-CdR treatment significantly reduced MeCP2 and DNMT3B occupancy in these CpG islands (Figure 6f and g), and administration of the miR-137 inhibitor rescued 5-Aza-CdR repression of ASCT2 expression (Supplementary Figure S6). Moreover, either MeCP2 depletion or DNMT inhibition, similar to miR-137 overexpression, significantly decreased glutamine consumption, α-KG production and ATP generation (Figure 7a–f). Altogether, these results suggest that MeCP2 and DNMTs cooperate to reactivate ASCT2 expression and glutamine metabolism through miR-137 epigenetic silencing.

## Discussion

Cancer cells exhibit increased metabolic autonomy in comparison to normal cells, importing and metabolizing nutrients required to support their characteristic unabated growth.^1, 2, 3, 4^ Glutamine is such an essential nutrient for cancer cells, serving as a carbon and nitrogen source for biosynthesis of macromolecules and, via conversion to α-ketoglutarate, as a source of metabolic intermediates to replenish the TCA cycle.^1, 2, 3, 4^ Thus, a better understanding of molecular mechanisms underlying glutamine metabolism should improve our knowledge to define their contribution to tumor initiation and/or progression.

Intracellular glutamine levels are strictly controlled by a group of membrane-anchored transporters. Since most tumor cells depend on increased glutamine metabolism, they must upregulate certain selective transporters to meet the high glutamine metabolic demands. Among which, ASCT2 is aberrantly elevated in multiple cancer types, frequently implicated in regulation of glutamine-dependent tumor cell growth.^18, 24, 37, 38^ Yet, genetic and/or epigenetic mechanisms underlying the control of ASCT2-mediated glutamine uptake are largely unknown.

Combined in-silico algorithms with systemic experimental screening, we herein identify the global tumor suppressor microRNA, miR-137, as an essential regulator that selectively targets ASCT2 and tumor glutamine metabolism. Our results suggest that epigenetic silencing of miR-137 by MeCP2 and DNMTs inversely enhanced ASCT2 translation and glutamine uptake (Figure 7g). These findings thus present a previously unreported mechanism depicting ASCT2 deregulation in human cancers. In addition to miR-137, the Myc family members, c-Myc in glioblastoma and N-Myc in neuroblastoma, directly activate *ASCT2* transcription and glutamine metabolism.^6, 23^ Most likely, these diverse mechanisms act in concert to specifically activate ACST2 expression in tumor cells.

It is particularly interesting why ASCT2 is selected and upregulated over other glutamine transporters in cancers given its narrow substrate selectivity and its mode of transport as an obligatory amino-acid exchanger.^18, 19^ Even though glutamine is a substrate, none of the essential amino acids except for threonine is the substrate of this transporter. Thus, how could this transporter coordinate tumor cell growth and proliferation? In addition to regulation of glutaminolysis to replenish the TCA cycle, part of the answer to this question appears to depend on the coupling of this transporter to SLC7A5 (also called LAT1), an important transporter responsible for essential amino-acid uptake, at the functional level. ASCT2 mediates the Na^+^-coupled influx of glutamine, and LAT1 then mediates the efflux of glutamine in exchange of the influx of essential amino acids (for example, isoleucine) and mTOR activation.^21, 22^ In this way, ASCT2 and LAT1 are co-opted to promote metabolic reprograming and aggressive tumor progression. In support of this notion, the expression levels of ASCT2 and LAT1 are coordinately upregulated in a wide spectrum of human cancers.^18, 19^

MiR-137 is frequently silenced during the process of oncogenesis, yet the mechanisms involved in remain to be clarified. We demonstrate that MeCP2 and DNMTs cooperate to promote active methylation of the miR-137 promoter leading to its transcriptional silencing. Moreover, we showed that, through downregulation of ASCT2 expression, miR-137 inhibits glutamine metabolism that is critical for cell proliferation and survival. Conceivably, miR-137 epigenetic silencing, together with additional essential oncogenic lesions (for example, Myc deregulation), would enable precancerous cells to alter their glutamine metabolism to survive and propagate in a stressed microenvironment, with the ‘fittest’ cells enduring and eventually acquiring cancerous properties.

Given that ASCT2 is frequently elevated in multiple cancer types and implicated in regulation of glutamine-dependent tumor cell growth, this transporter represents a potential drug target in cancer therapy. Due to a lack of high-affinity, specific small chemical inhibitors or monoclonal therapeutic antibodies, direct targeting of ASCT2 is currently not applicable. In this regard, our results suggest that DNMT inhibitors can be used as an alternative approach to indirectly targeting ASCT2 (via miR-137 reactivation) and glutamine metabolism. As DNMT inhibitors have been approved for patient treatment, our study thus prompts immediate clinical trial evaluation of these inhibitors, alone or in combination, in cancer patients exhibiting ASCT2/glutamine addiction.

In conclusion, our findings not only identify a novel mechanism depicting the miR-137 tumor suppressor functions but also provide a mechanistic basis for differences in glutamine metabolism, a key driver of tumor development and response to therapy.

## Materials and methods

### Cell culture

BE-2C cells were kindly provided by Dr Michael D Hogarty at Children’s Hospital of Philadelphia, University of Pennsylvania, and maintained in RPMI media (GIBCO) containing 10% fetal bovine serum, 2 mM glutamine and 1 × penicillin and streptomycin. NCM460 and remaining tumor cells were maintained in Dulbecco’s modified Eagle’s medium supplemented with 10% fetal bovine serum, 2 mM glutamine and 1 × penicillin and streptomycin. All the cells used in the experiments were validated as Mycoplasma-negative.

### Plasmid construction

Pre-miR-137 and specific shRNAs against ASCT2, MeCP2 and GFP were cloned into pLKO.1 lentiviral vector with synthesized DNA oligos. ASCT2 open reading frame was amplified by specific primers and cloned to pCDH-CMV-GFP vector (System Biosciences, Johnstown, PA, USA). All the DNA oligo and primer sequences were shown in the Supplementary Tables 1.

### RNA extraction and real-time qPCR

Total RNA was isolated from cells using TRIzol (Ambion, Austin, TX, USA) according to the manufacture’s protocol. Reverse transcription was performed using Reve Tra Ace qPCR RT kit (Toyobo, Kita-Ku, Osaka, Japan). MiRNAs were purified with miniRNA fast extraction kit (Aidlab, Beijing, China), and reverse transcription was performed using Taqman MicroRNA Reverse Transcription Kit (Thermo Fisher Scientific, Waltham, MA, USA). Relative mRNA levels were quantified by SYBR Green-based real-time qPCR with gene-specific primers shown in the Supplementary Tables 1. Relative expression of protein-coding genes were normalized to that of actin. Relative miR-137 levels were assayed with Taqman specific primers (Thermo Fisher Scientific) and normalized to that of 18 S.

### Western blot

Cells or fresh colorectal carcinoma specimens were lysed in RIPA buffer (50 mM Tris-HCl, pH 7.4, 150 mm NaCl, 1 mM EDTA, 0.25% sodium deoxycholate, 1% Nonidet P-40, 1 mM dithiothreitol, 1 mM phenylmethylsulfonyl fluoride). Fifty microgram total cellular lysates were used for each blot. The antibodies used were as follows: ASCT2 (#8057, Cell Signaling Technology, Danvers, MA, USA), GLS1 (Abcam, ab60709), MeCP2 (ab2828, Abcam, London, UK) and Actin (sc-47778, Santa Cruz, Dallas, TX, USA).

### Luciferase reporter assay

Potential miR137-binding site (wildtype or mutant, see Supplementary Table S1) in the 3′-UTR of human *ASCT2* were synthesized from GENEWIZ Inc., China and cloned into the *Xba*I site of pGL3-promoter vector (Promega, Madison, WI, USA). Luciferase activities were measured 24 hr posttransfection with a Dual Luciferase Kit (Promega). Firefly luciferase activities were normalized to Renilla luciferase control values and shown as an average of triplicates.

### Gas chromatography mass spectrometry

HCT116 cells were infected with a control or ASCT2 shRNA and selected with puromycin for 48 h, and then switched to fresh medium for another 24 h. Cells were rinsed with 1 ml ice-cold PBS and quenched with 1 ml 80% methanol (methanol: water=4:1, v/v). Cells were collected in tubes by scraping with a pipette and the extracts were vortexed for 5 min. Samples were centrifuged at 14 000 *g* for 15 min, and the supernatant was transferred to a new tube for evaporation in a CentriVap Centrifugal Vacuum Concentrator (Labconco, Kansa, MO, USA). The dried metabolites were dissolved in 50 μl methoxyamine pyridine (20 mg/ml), vortexed for 30 s, and ultrasound-treated for 10 min. Oximation was then conducted at 40 °C for 2 hr, followed by silylation with 40 μl MSTFA for 1 hr. After derivatization, the solution was centrifuged at 14 000 g for 15 min and diverted to a 2 ml glass vial. Metabolic profiling was performed by GCMS-QP 2010 analytical system (Shimadzu, Tokyo, Japan) as previously described.^39^

### Representative metabolites analysis

HCT116 cells with ASCT2 depletion or miR-137 overexpression were cultured in Dulbecco’s modified Eagle’s medium media with 2 mM glutamine for 24 h. Glutamine consumption, α-KG and ATP contents were determined using respective assay kits obtained from BioVision (Milpitas, CA, USA). Data are an average of triplicate and presented as a percentage of the control group.

### ChIP assay

ChIP was performed as previously described.^40^ The primers used were described in Supplementary Table S1. The antibodies used in ChIP were as follows: MeCP2 (ab2828, Abcam), DNMT1 (ab13537, Abcam), DNMT3B (ab2851, Abcam) and IgG (sc-2025, Santa Cruz).

### Xenografts in nude mice

BALB/C nude mice (4–6 weeks old) were injected subcutaneously in both flanks with 4 million HCT116 cells expressing the control vector, ASCT2, miR-137 or miR-137/ASCT2 combination in 200 μl PBS. Tumor volumes were measured every 3 days, and tumor weight was measured at the time of sacrifice. All animal experiments were performed following the animal guidelines of university laboratory and with approval from the Animal Experimentations Ethics Committee of Tongji Medical College, Huazhong University of Science & Technology.

### Human specimens

Fresh de-identified human colorectal tumor samples were obtained from the Union Hospital, Tongji Medical College, with patient consent and Institutional Review Board Approval. The procedures involving human subjects were in accordance with the Helsinki Declaration.

### Immunohistochemistry

Tumor sections from mouse xenografts were incubated with the antibodies against control IgG, ASCT2 (HPA035240, Sigma, St Louis, MO, USA) and PCNA (Santa Cruz, sc-56) overnight at 4 °C. The remaining steps were performed using the DAKO CSA kit (Carpinteria, Santa Clara, CA, USA).

### Statistical analysis

Data were presented as mean±s.d. from at least three independent experiments. The correlation between miR-137 and *ASCT2* in TCGA data sets was performed by Pearson’s correlation. All the remaining significance analysis were performed using two-tailed Student’s *t*-test.
