# Supplementary material for: Epigenetic silencing of microRNA-137 enhances ASCT2 expression and tumor glutamine metabolism
Source: Oncogenesis. 2017 Jul 10;6(7):e356–. doi: 10.1038/oncsis.2017.59 (PMC5541711; doi:10.1038/oncsis.2017.59)
Supplement: Supplementary Information [file oncsis201759x1.docx]

**Supplementary Information**

**
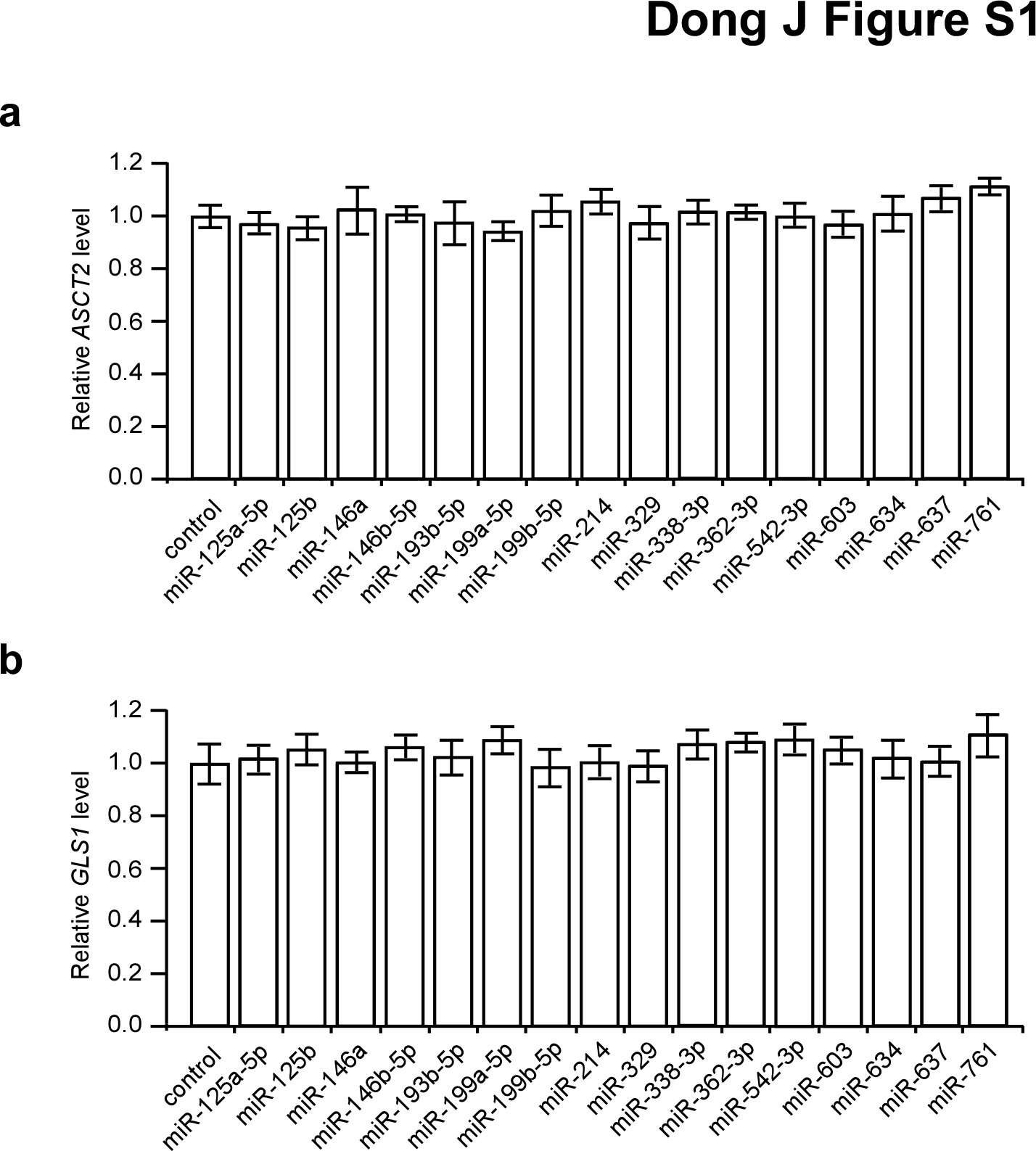
**

**Supplementary Figure 1.** Real-time qPCR quantification of *ASCT2* (a) and *GLS1* (b) mRNA levels in 293T cells 24 hr post transfection of control or indicated miRNA mimics.


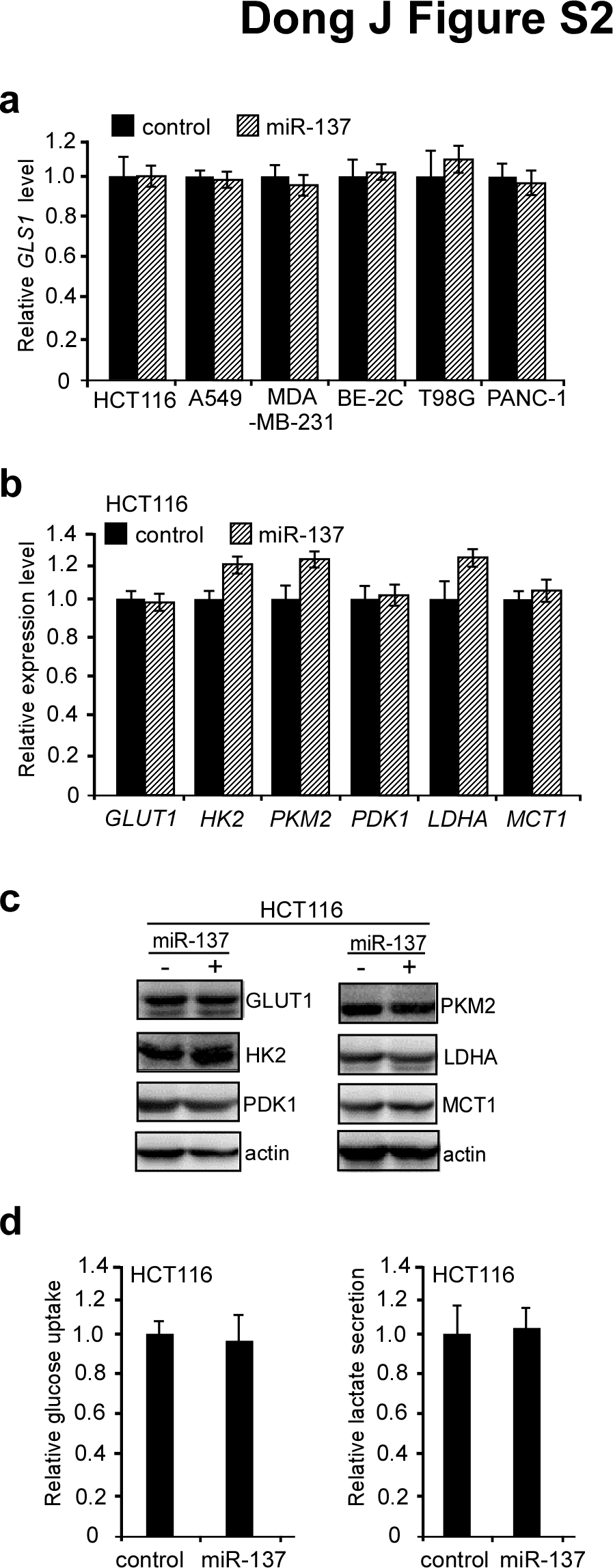


**Supplementary Figure 2.** (a) MiR-137 mimics had minimal effect on *GLS1* mRNA levels in tumor cells from different cancer types. (b-c) Changes in mRNA (b) and protein (c) levels of glycolytic genes upon transfection of miR-137 mimics. (d) Changes in glucose uptake and lactate secretion in HCT116 cells transfected with control or miR-137 mimics.


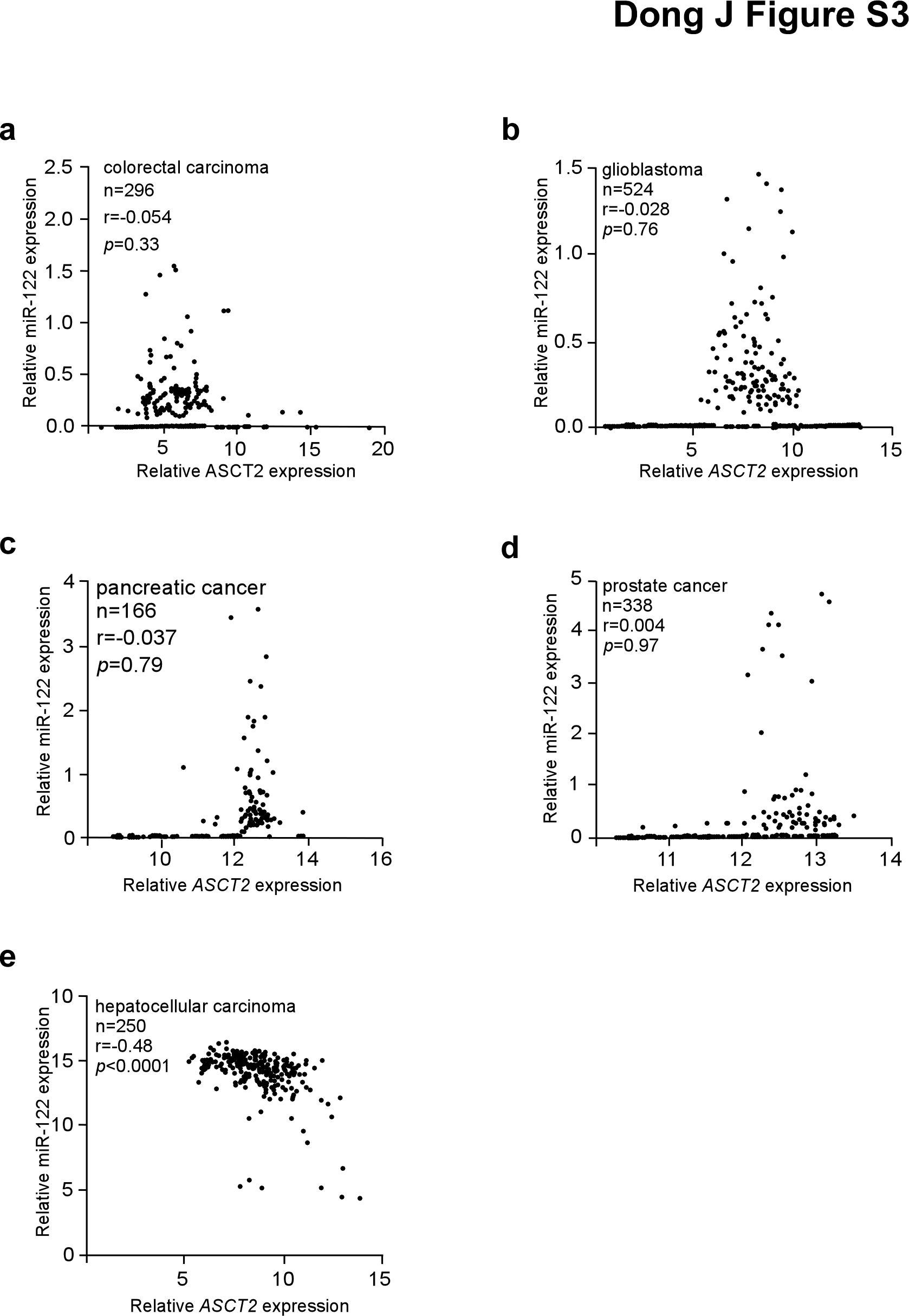


**Supplementary Figure 3.** Correlation between miR-122 and *ASCT2* in colorectal carcinoma (a), glioblastoma (b), pancreatic cancer (c), prostate cancer (d) and hepatocellular carcinoma (e).


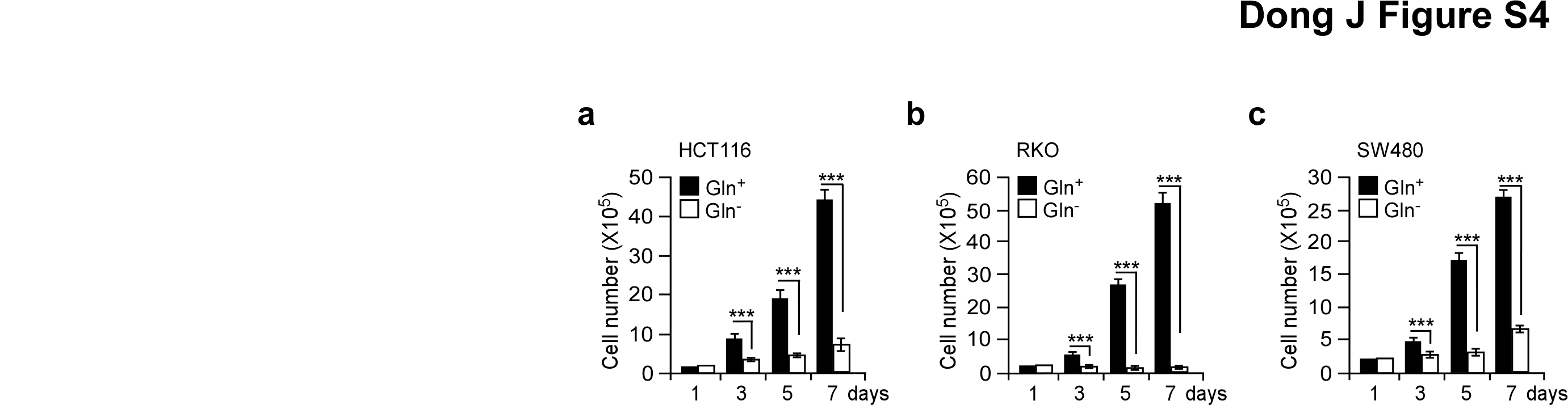


**Supplementary Figure 4.** Proliferation of HCT116, RKO and SW480 cells in the presence or absence of glutamine, as measured by serial cell counts over 7 days. Mean ± SD (n = 3), t-test; ***p<0.001.


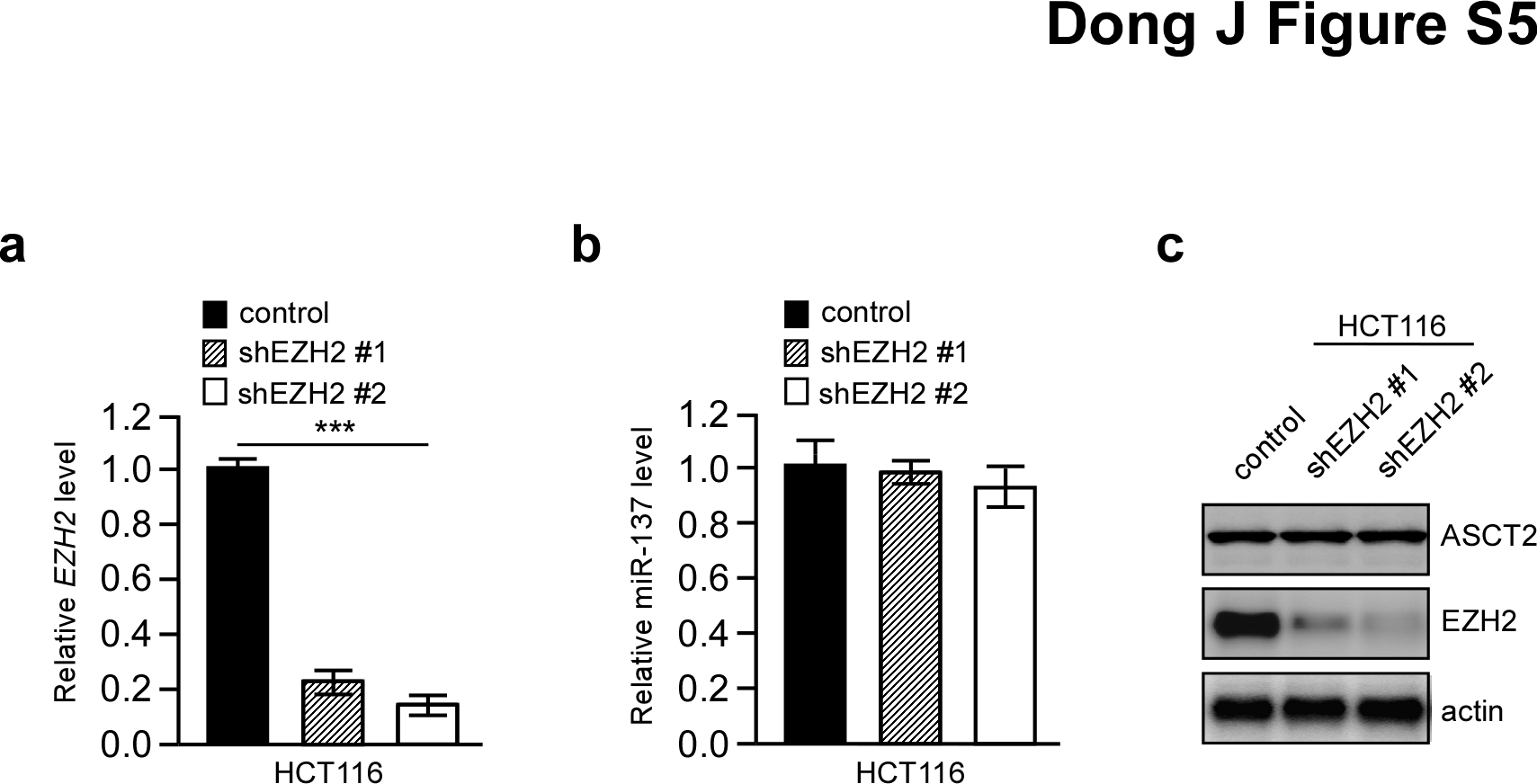


**Supplementary Figure 5.** Real-time qPCR analysis of *EZH2* (a) and miR-137 (b) levels upon depletion of EZH2 expression in HCT116 cells. (c) Immunoblot detection of ASCT2 levels upon *EZH2* knockdown. Actin was used as a loading control. Mean ± SD (n = 3), t-test; ***p<0.001.


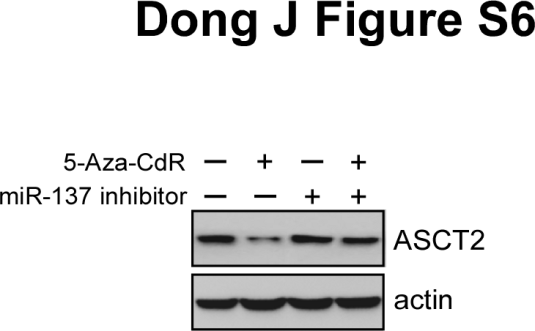


**Supplementary Figure 6.** Administration of miR-137 inhibitor rescued ASCT2 expression suppressed by the DNMT inhibitor 5-Aza-CdR. HCT116 cells were pre-treated with DMSO (control solution) or 5-Aza-CdR (10 µM) for 48 hr, then transfected with a mock or miR137 inhibitor (200 nM) in the presence or absence of 10 µM 5-Aza-CdR for additional 48 hr. Cells were then harvested and ASCT2 expression were analyzed by immunoblot with actin as a loading control.

**Supplementary Table 1. Primer sequences used in the study**

| primer name | sequence (5' to 3') |
| --- | --- |
| qPCR primers |  |
| ASCT2 F | CGGCACGCCCGGGAGGCTTTC |
| ASCT2 R | GAATCTGGGGGCCGGGAAGCGG |
| β-actin F | AGCCTCGCCTTTGCCGA |
| β-actin R | GCGCGGCGATATCATCATC |
| 18S-F | GAATTCCCAGTAAGTGCGGG |
| 18S-R | GGGCAGGGACTTAATCAACG |
|  |  |
| Primers for ChIP |  |
| Primer set 1F | CATTTGGATTTGGGCAGGAAGC |
| Primer set 1R | GAACTCTTGCTGCTCGCTGA |
| Primer set 2F | TCAGCGAGCAGCAAGAGTTC |
| Primer set 2R | CCAAGAATACCCGTCACCGAAG |
| Primer set 3F | TCCTCTGACTCTCTTCGGTGAC |
| Primer set 3R | CTTGGCAACCACGGGCGTTTAG |
|  |  |
| Primers for cloning |  |
| pre-miR-137 F | CCGGTGGTCCTCTGACTCTCTTCGGTGACGGGTATTCTTGGGTGGATAATACGG ATTACGTTGTTATTGCTTAAGAATACGCGTAGTCGAGGAGAGTACCAGCGGCAG |
| pre-miR-137 R | AATTCTGCCGCTGGTACTCTCCTCGACTACGCGTATTCTTAAGCAATAACAACG  TAATCCGTATTATCCACCCAAGAATACCCGTCACCGAAGAGAGTCAGAGGACCA |
| ASCT2-wt-3'UTR F | CTAGAGAGGACATTTTTTTTAGCAATAAGAATTCGAGGACATTTTTTTTAGCAATAA |
| ASCT2-wt-3'UTR R | CTAGATTATTGCTAAAAAAAATGTCCTCGAATTCTTATTGCTAAAAAAAATGTCCTCT |
| ASCT2-mut-3'UTR F | CTAGAGAGGACATTTTTTTTCTAGCACAAGAATTCGAGGACATTTTTTTTCTAGCACAT |
| ASCT2-mut-3'UTR R | CTAGATTGTGCTAGAAAAAAAATGTCCTCGAATTCTTGTGCTAGAAAAAAAATGTCCTCT |
| ASCT2-ORF F | GCTCTAGAGCCACCATGGTGGCCGATCCTCCTCG |
| ASCT2-ORF R | CGGAATTCCATGACTGATTCCTTCTCAGAG |
